# Correlates of Heart Rate Measures with Incidental Physical Activity and Cardiorespiratory Fitness in Overweight Female Workers

**DOI:** 10.3389/fphys.2015.00405

**Published:** 2016-01-07

**Authors:** Laís Tonello, Felipe F. Reichert, Iransé Oliveira-Silva, Sebastián Del Rosso, Anthony S. Leicht, Daniel A. Boullosa

**Affiliations:** ^1^Departamento de Educação Física, Centro Universitário UnirGGurupi, Brazil; ^2^Escola Superior de Educação Física, Universidade Federal de PelotasPelotas, Brazil; ^3^Departamento de Educação Física, UniEVANGÉLICA-Centro Universitário de AnápolisAnápolis, Brazil; ^4^Departamento de Educação Física, Universidade Católica de BrasíliaÁguas Claras, Brazil; ^5^Sport and Exercise Science, James Cook UniversityTownsville, QLD, Australia

**Keywords:** heart rate variability, heart rate recovery, cardiorespiratory fitness, incidental physical activity, females, work, allostatic load, autonomic nervous system

## Abstract

Previous studies have suggested that physical activity (PA) levels and cardiorespiratory fitness (CRF) impact on the autonomic control of heart rate (HR). However, previous studies evaluating PA levels did not discriminate between incidental PA and regular exercise. We hypothesized that incidental PA “*per se*” would influence cardiac autonomic indices as assessed via HR variability (HRV) and HR recovery (HRR) in non-exercisers. Thus, the objective of this study was to investigate the relationships between objective PA levels, CRF, and cardiac autonomic indices in adult, regular non-exercising female workers. After familiarization with procedures and evaluation of body composition, 21 women completed a submaximal cycling test and evaluation of HRR on four different days. Resting (2-min seated and standing) and ambulatory (4-h) HRV were also recorded. Levels of PA were assessed by accelerometry over five consecutive days (i.e., Wednesday to Sunday). Maximum oxygen consumption (VO_2_max) was measured as an index of CRF. As reliability was low to moderate for most HR measures, relationships between these and PA and CRF were examined using the 4-day average measures. Significant correlations were identified between post-exercise HRR in the first min with various PA indices (daily moderate PA, daily vigorous PA, and the sum of vigorous and very vigorous daily PA). Additionally, VO_2_max was significantly correlated to HRV but not to HRR. The current results indicated that CRF was influential in enhancing HRV while incidental or non-exercise based PA was associated with greater autonomic reactivation in adult overweight women. Therefore, both CRF and non-exercise based PA contribute significant but diverse effects on cardiac health. The use of 4-day averages instead of single measures for evaluation of autonomic control of HR may provide a better indication of regular cardiac autonomic function that remains to be refined.

## Introduction

Regular exercise and physical activity (PA) contribute to human health and its maintenance (Kruk, 2007; Garber et al., 2011) in similar ways for both men and women (Schumann et al., 2015). While exercise refers to structured repetitive movements that are planned for improving or maintaining physical fitness, PA refers to any body movement produced by muscular activity which results in energy expenditure above resting levels (Ainsworth et al., 2000). Undertaking regular aerobic exercise and maintaining appropriate levels of PA are simple and low-cost interventions for the improvement of cardiorespiratory fitness (CRF), a factor strongly linked to the incidence and risk of most cardiometabolic diseases (LaMonte et al., 2005; Jae et al., 2007; Jakicic et al., 2009). That is, high levels of CRF are related to lower rates of mortality and morbidity among individuals, especially those with cardiovascular disease (Lee et al., 1999, 2010; Wei et al., 1999). Further, low CRF has been reported to affect health more negatively in women compared to men (Skaug et al., 2014), therefore, gender differences may be of key importance when examining CRF and cardiovascular health.

Like the relationship between CRF and cardiovascular health, greater cardiac autonomic control, specifically enhanced parasympathetic and reduced sympathetic activity, has been associated with lower rates of mortality and morbidity in a range of chronic conditions (Nolan et al., 1998; La Rovere et al., 2001; Stein et al., 2008; Pei et al., 2015). This relationship may also be a resultant of greater CRF with improvements in cardiac autonomic activity coinciding with increases in CRF following chronic exercise training (Leicht et al., 2003; Kiviniemi et al., 2010). Previously, Davy et al. (1998) found that cardiac autonomic control and cardiac baroreflex sensitivity decline similarly with age in healthy sedentary and physically active women, however, physically active women demonstrate higher levels of cardiac autonomic control and cardiac baroreflex sensitivity compared with their sedentary peers, regardless of age. All of these factors (PA, CRF, and cardiac autonomic control) have strong effects on health (Ramsbottom et al., 2010). However, the interplay between these factors has not been fully elucidated, possibly as a result of different methods employed to evaluate cardiac autonomic control.

Heart rate variability (HRV) is a simple and widely utilized non-invasive method for evaluation of cardiac autonomic control during basal, orthostatic, and ambulatory conditions (Task Force, 1996). Additionally, post-exercise HR recovery (HRR) has been widely utilized as a simple measure of cardiac autonomic control, particularly parasympathetic reactivation (Cole et al., 1999; Boullosa et al., 2009; Gordon et al., 2011; Daanen et al., 2012). Both HRV and HRR have been extensively utilized in different settings (Gordon et al., 2011; Uusitalo et al., 2011; Boullosa et al., 2012) with a variety of methodological constraints such as reliability, body posture, duration and number of recordings, and parameters selected (Young and Leicht, 2011; Boullosa et al., 2013, 2014; Plews et al., 2014). For example, previous studies have indicated that HRV during the monitoring of training was related to CRF (Kiviniemi et al., 2007; Hautala et al., 2009) while HRR was influenced by the applied exercise load (Buchheit and Gindre, 2006; Guerra et al., 2014). More recently, studies within sport settings (Plews et al., 2012, 2014; Boullosa et al., 2013) have suggested the need for multiple HRV measures (e.g., 3–4 weekly measures; Boullosa et al., 2013; Plews et al., 2014) instead of isolated single measures for a better evaluation of autonomic adaptations. Consequently, it is possible that variable selection for cardiac autonomic control measure, along with frequency of assessment (i.e., 1 vs. >1 recording) may have a substantial impact upon its relationship with other health measures (e.g., PA or CRF). This is especially important in light of the variable reliability values reported for HRV measures during different conditions such as ambulatory (Myrtek, 1990; Ziegler et al., 1999) and following sub-maximal and maximal exercise testing (Arduini et al., 2011; Dupuy et al., 2012; Boullosa et al., 2014).

Similarly, the assessment of PA via different tools (e.g., accelerometers, questionnaire) may also influence its relationship with health indicators (Ara et al., 2015). Most studies to date have evaluated PA levels by questionnaires which are vulnerable to bias (Lindholm et al., 2012; Pavey et al., 2013; Ara et al., 2015). Consequently, objective tools (e.g., accelerometers) have been increasingly utilized to document PA levels (Buchheit et al., 2005, 2006; Hansen et al., 2011). While these devices provide a more precise indication of PA levels, particularly the intensity of the PA, very few studies have differentiated between structured exercise and incidental PA (i.e., non-purposeful PA accrued through activities of daily living; Ross and McGuire, 2011). Therefore, the positive relationships between objective PA and health indicators (e.g., CRF) may be a result of incidental PA and exercise undertaken. Recently, incidental PA was reported to influence CRF improvements during an exercise intervention with recreational athletes (Hautala et al., 2012) while the duration and intensity of incidental PA was positively correlated with CRF in obese individuals (Ross and McGuire, 2011). Therefore, examinations between objective measures of PA and other health indicators (e.g., CRF, HRV) should account for incidental PA and exercise.

Given the significant relationships noted between cardiac autonomic control, CRF and PA, and the impact of these for improved health, further examination of the interaction between these variables was required for clarification. Thus, the objective of the current study was to evaluate the relationship between different cardiac autonomic measures (HRV, HRR), and CRF and objective PA levels in overweight but healthy female adults.

## Material and methods

### Participants

Twenty-one young, non-menopausal, overweight but healthy women, free from pathological conditions (i.e., diabetes, hypertension, cardiovascular disease, depression, etc.) and medications that could interfere with the outcome measures, volunteered for this study. All participants were full-time service workers (e.g., cleaning and administrative positions) of the Catholic University of Brasilia and were not undertaking any structured exercise regime at the time of the study. The inclusion of non-exercising participants enabled a better isolation of the effects of incidental PA levels on health related parameters. All participants performed all procedures and adhered to similar work schedules during the day (i.e., work day beginning at 7 a.m.). The ethical committee of the Catholic University of Brasilia approved this study and all participants provided informed written consent before participation.

### Study design

**Figure 1:**
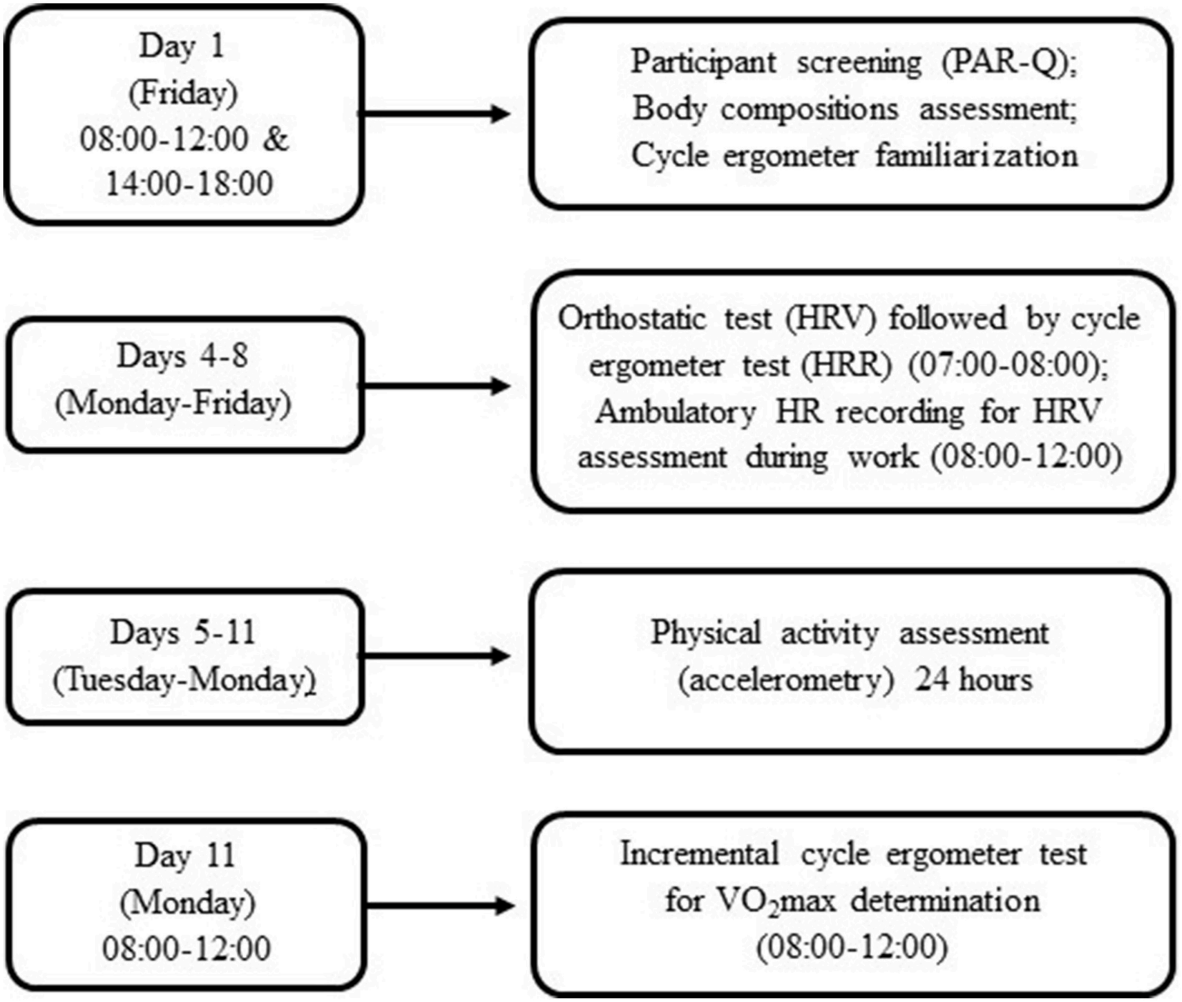
**Study design**. Par-Q, Physical Activity Readiness Questionnaire; HRV, Heart Rate Variability; HRR, Heart Rate Recovery; HR, Heart Rate; VO_2_max, Maximum oxygen consumption.

This study was conducted over an 11-day period (Figure 1) following a familiarization session (1-week earlier). On Day 1 (i.e., Friday), participants were screened, familiarized with all procedures and assessed for body composition. During Days 4–8 (i.e., Monday–Friday), participants visited the laboratory each morning and completed an orthostatic test and a constant-load cycling exercise bout for the determination of HRV and HRR measures. Following this bout, an ambulatory 4-h R-R recording was obtained from each participant during working hours and assessed for ambulatory HRV. Participants' PA levels were recorded over 7 days (i.e., from Tuesday to Monday) of the study. On the final day (i.e., Monday, day 11), participants performed an incremental cycle ergometer test for the determination of maximum oxygen consumption (VO_2_max), an index of CRF.

### Anthropometry

Body mass (kg) was evaluated using a digital scale (G-Tech, 05, China®) and height (cm) assessed via a stadiometer (Sanny®, ES2040, São Bernardo do Campo, Brazil) for determination of body mass index (BMI). Waist circumference was determined at the smallest girth of the trunk using a metal anthropometric tape (Sanny, SN4010, Medical®, Brazil). Body composition (% fat) was assessed from skinfold measurements obtained using skinfold calipers (Lange Skinfold Caliper® California, USA) in accordance with Jackson and Pollock (1985). Three measurements were made at the following sites: triceps, subscapular, abdominal, axillary, thigh, chest, and suprailiac. Body fat composition (%) was calculated from the average of the three measurements at each skinfold site using the Siri equation (Siri, 1961).

### Heart rate variability

Assessment of HRV was conducted in two stages, an orthostatic test and during normal work activities (08:00–12:00). The daily orthostatic test was performed in the laboratory (07:00–08:00) within standard environmental conditions. The orthostatic test consisted of participants sitting for 3 min followed by 4 min of standing. All analyses were conducted during the last 2 min of each position. Following the orthostatic and submaximal exercise tests (see below), participants left the laboratory and undertook their normal, morning (08:00–12:00) work activities while wearing a telemetric HR monitor (see below). Assessment of daily HRV during work was determined from the entire 4-h HR recording.

All HR recordings were obtained using a HR monitor (RS800CX, Polar Electro Oy, Finland) reported to provide valid recordings for HRV assessment (Wallén et al., 2012) at a sampling rate of 1000 Hz. Recordings were uploaded into a computer and filtered using the manufacturers' software (Polar ProTrainer® version 5.0, POLAR Electro Oy, Kuopio, Finland) followed by exportation to a dedicated program (Kubios HRV v2.0, Kuopio University, Finland) for the HRV analyses. The HRV variables examined included those previously examined in other studies of exercise and HRV (Leicht et al., 2011; Boullosa et al., 2014): time domain (SDNN and RMSSD), frequency domain (LF and HF in both absolute and normalized units), and non-linear measures (SD1, SD2, Sample Entropy, and α1) (Task Force, 1996; Acharya et al., 2004; Boullosa et al., 2014). Based on previous studies (Boullosa et al., 2013; Plews et al., 2014), daily HRV measures for each of the 4 days (from Tuesday to Friday) as well as the average of the 4 days were used for further analyses.

### Heart rate recovery

A submaximal, square-wave, exercise bout was undertaken by participants each day over 4 consecutive days and utilized for the determination of HRR. Participants exercised on a cycle ergometer (Monark model 8348, Monark, Sweden) at 60 rpm for 6 min (Arduini et al., 2011) with the workload increased every 30 s for the first 3 min to achieve a target HR and thereafter remained constant for the exercise bout. The set target was a workload that induced a HR of ~86% of age-predicted maximum HR (HR_max,_ Tanaka et al., 2001) with the target HR and protocol determined during the familiarization session to ensure a consistent exercise response. This target HR was suggested to provide the most reliable HRR measures (Lamberts et al., 2011).

The change (Δ) in HR during the first 1, 2, 3, and 5 min of recovery were evaluated as HRR measures (Arduini et al., 2011). Additionally, the HRR index, as proposed by Imai et al. (1994), was calculated via semi-logarithmic regression. Briefly, the natural logarithm of the instantaneous HR during the initial rapid HR decrease (from the 10th to the 40th s) was plotted against the elapsed time of recovery and a linear regression analysis was applied. The time constant of the short, post-exercise HR decay (T30) was thus determined as the negative reciprocal of the slope of the regression line. Following the same rationale for the HRV measures (Boullosa et al., 2013), the daily HRR measures (Imai et al., 1994; Arduini et al., 2011), as well as the average HRR for the 4 monitoring days were utilized for analyses.

### Physical activity assessment

PA was objectively measured by an accelerometer (GT1M, Actigraph, USA) for 7 consecutive days. The devices were used on the right hip of participants and recorded continuously from Tuesday to the next Monday, except during the bathing, sleeping and the cycle ergometer evaluations.

The start and end days of the accelerometer recording (i.e., data from Tuesday and Monday) were excluded with the final analysis of PA comprising 5 full days (i.e., Wednesday to Sunday only). Three to five days of PA assessment have been suggested to be sufficient to accurately reflect weekly PA patterns of adults (Trost et al., 2005). Technical details of this accelerometry device and its measurement of PA intensity have been published elsewhere (John and Freedson, 2012). Briefly, these devices measure accelerations in the vertical plane at a sampling frequency (epoch) of 5 Hz, which is preferable over longer epochs (example 60 s) (Orme et al., 2014). The unit of measurement of the accelerometer was counts per minute with higher counts per minute indicating greater accelerations and intensity of the activity. In the present study, the well-established (Freedson et al., 1998) cut-off limits were chosen to determine moderate (1952 counts/min), vigorous (5725 counts/min), and very vigorous (>9499 counts/min) PA categories. These cut-off limits represent PA intensities of 3, 6, and >8.99 METS, respectively (Freedson et al., 1998). The accumulated time spent (minutes per day) in physical activities for each of these PA categories was calculated.

In addition to PA intensity, step count was also included in the analysis. The GT1M includes a step counting mode which records the number of positive accelerations followed immediately by a negative acceleration (i.e., steps and steps/day) undertaken by the user. This measure was included as an easily interpretable indicator of overall volume of PA (Tudor-Locke et al., 2011).

### Cardiorespiratory fitness

Cardiorespiratory fitness was assessed as the VO_2_max during a maximal incremental test on a cycle ergometer (Lode Excalibur, Lode, Netherlands; or Monark model 8348, Monark, Sweden). The test started with a load of 0 W and thereafter the load was increased at a rate of 20 W·min^−1^, maintaining a constant cadence of 60 rpm. Throughout the graded exercise test, HR was recorded using a telemetric monitor (POLAR Electro Oy, Finland) while ventilatory parameters (e.g., oxygen consumption, VO_2_) were assessed breath-by-breath via a metabolic cart (Metalyzer 3B, Cortex, Leipzig, Germany). All participants were verbally encouraged to exercise until voluntary exhaustion with HR similar to or greater than age-predicted HR_max_ (Tanaka et al., 2001) and respiratory exchange ratio greater than 1.1 (Howley et al., 1995) defining VO_2_max.

### Statistical analysis

Statistical analysis was performed with a statistical package (SPSS, v 20.0, IBM). Descriptive statistics were used to present means, standard deviations (±SD) and 90% confidence interval (90% CI). Normality was assessed by Shapiro-Wilk test. Variables with non-normal distribution were log-transformed (Ln) for analysis but presented in original units. As varying degrees of reliability have been reported for HR measures during various conditions (Arduini et al., 2011; Young and Leicht, 2011; Dupuy et al., 2012; Boullosa et al., 2014), reliability for HRV and HRR measures were assessed via typical error of measurement (TEM) expressed as the coefficient of variation (CV, %) for absolute reliability and intra-class correlation coefficient (ICC) for relative reliability (i.e., ratio of variance due to differences between subjects to the total variability in the data) (Hopkins, 2000a). Reliability measures were calculated using a reliability spread-sheet (Hopkins, 2000b) with known thresholds for varying levels of reliability. For the HRR kinetics, data were modeled with a monoexponential fit (Sigmaplot 12; SPSS Science, Chicago, IL) as previously described (Boullosa et al., 2014) with the time constant (τ) used for further analysis. Pearson product correlation coefficients (r) with 90% confidence intervals (90% CI) were calculated to assess the relationships between selected parameters. The level of significance was set at *p* < 0.05.

## Results

Demographic characteristics and PA levels of participants are shown in Table 1. Briefly, all females were overweight (BMI>25), with low CRF, undertook an average of >10,000 steps/day and were engaged mainly in moderate levels of PA.

**Table 1:** **Demographic characteristics and physical activity levels of participants (***n*** = 21)**.

**Parameters**	**Mean (*SD*)**
Age (years)	34.5 (6.4)
Height (m)	1.60 (0.06)
Weight (kg)	67.0 (11.37)
BMI (kg/m^2^)	26.3 (4.1)
% Fat	37.0 (4.7)
WC (cm)	79.7 (9.7)
VO_2_max (ml·kg^−1^·min^−1^)	24.6 (5.3)
Steps·day^−1^	10424 (3047)
MPA (min/day)	57.22 (18.23)
VPA (min/day)	1.16 (0.93)
VVPA (min/day)	0.08 (0.11)
VPA + VVPA (min/day)	1.24 (0.96)

*SD, Standard Deviation; BMI, body mass index; WC, waist circumference; VO_2_max, Maximum oxygen consumption. PA variables are presented as a daily mean for the 5 days of data collection; Steps·day^−1^, mean daily steps; MPA, mean daily moderate physical activity; VPA, mean daily vigorous physical activity; VVPA, mean daily very vigorous physical activity; VPA + VVPA, sum of vigorous physical activity and very vigorous physical activity*.

Seated and standing HRV measures during the orthostatic test, ambulatory HRV measures, and HRR measures on the 4 different days are presented in Tables 2A,B, 3, 4, respectively. Average reliability for the HRV measures during seated rest (see Table 2A) was moderate while low to moderate during orthostatic stress (see Table 2B). Similarly, the HRV ambulatory measures exhibited variable reliability from poor to excellent (see Table 3). For the HRR measures, reliability was low to moderate (see Table 4). As reliability was low to moderate for most HR measures, relationships between these and other variables were examined using the 4-day average measures.

**Table 2A:** **Heart rate variability measures seated during the orthostatic test and their corresponding reliability measures**.

	**Tuesday**	**Wednesday**	**Thursday**	**Friday**	**Average**	**CV, %**	**ICC**
SDNN (ms)	38.9 ± 13.0 (34.2–43.5)	42.1 ± 12.0 (37.8–46.4)	42.7 ± 12.0 (38.4–47.0)	42.6 ± 17.6 (36.3–48.9)	41.5 ± 11.3 (37.5–45.6)	8.6 (7.4–10.5)	0.63 (0.45–0.79)
RMSSD (ms)	26.1 ± 13.2 (21.3–30.8)	27.0 ± 12.7 (22.4–31.5)	24.5 ± 9.2 (21.1–27.8)	29.8 ± 17.7 (23.4–30.3)	26.8 ± 9.8 (23.3–30.3)	10.4 (9.0–12.7)	0.43 (0.22–0.64)
LF (ms)^2^	520 ± 398 (377–663)	540 ± 463 (373–706)	611 ± 580 (403–820)	777 ± 671 (553–926)	615 ± 456 (451–779)	310 (267–378)	0.69 (0.52–0.82)
LF (n.u.)	65.48 ± 23.79 (56.94–74.02)	64.37 ± 23.15 (56.06–72.68)	68.39 ± 20.14 (61.16–75.62)	69.71 ± 23.11 (61.41–78.00)	66.99 ± 18.83 (60.23–73.75)	14.42 (12.45–17.60)	0.32 (0.12–0.55)
HF (ms)^2^	324 ± 322 (208–439)	291 ± 278 (191–391)	267 ± 214 (190–344)	451 ± 663 (213–689)	333 ± 289 (229–437)	281 (243–343)	0.55 (0.35–0.73)
HF (n.u.)	34.51 ± 20.42 (27.18–41.85)	35.63 ± 19.99 (28.45–42.80)	31.60 ± 15.18 (26.15–37.05)	30.28 ± 18.78 (23.54–37.03)	33.00 ± 19.93 (28.00–38.01)	14.42 (12.45–17.60)	0.32 (0.12–0.55)
SD1 (ms)	19.7 ± 8.9 (16.4–22.8)	20.0 ± 10.3 (16.3–23.7)	16.5 ± 7.3 (13.8–19.1)	20.8 ± 12.6 (16.3–25.3)	19.2 ± 13.0 (16.6–21.8)	7.6 (6.5–9.2)	0.43 (0.23–0.64)
SD2 (ms)	53.4 ± 15.7 (47.8–59.1)	57.8 ± 16.8 (51.8–63.8)	54.0 ± 16.8 (47.9–60.0)	58.7 ± 24.2 (50.0–67.3)	56.0 ± 15.6 (50.3–61.5)	11.7 (10.1–14.2)	0.63 (0.54–0.76)
α1	1.13 ± 0.30 (1.02–1.24)	1.24 ± 0.28 (1.14–1.34)	1.21 ± 0.17 (1.15–1.28)	1.25 ± 0.27 (1.16–1.36)	1.21 ± 0.27 (1.14–1.28)	20.3 (17.5–24.8)	0.43 (0.22–0.64)
SampEn	1.42 ± 0.35 (1.29–1.55)	1.25 ± 0.40 (0.72–2.26)	1.34 ± 0.35 (1.25–1.50)	1.47 ± 0.30 (1.36–1.57)	1.38 ± 0.35 (1.23–1.90)	0.98 (0.84–1.19)	0.05 (−0.12–0.28)

*Values are mean ± SD (confidence intervals, CI 90%); standard deviation of all R-R intervals (SDNN), root mean square of successive differences between normal sinus R-R intervals (RMSSD), low-frequency (LF), high-frequency (HF), Very-low frequency (VLF), Total power, short-term beat-to-beat R-R variability from the Poincaré plot (SD1), long-term beat-to-beat variability from the Poincaré plot (SD2), and detrended fluctuations of short fractal scaling (α1), sample entropy (SampEn), TEM expressed as the coefficient of variation (CV%), intra-class correlation coefficient (ICC)*.

**Table 2B:** **Heart rate variability measures standing during the orthostatic test and their corresponding reliability measures**.

	**Tuesday**	**Wednesday**	**Thursday**	**Friday**	**Average**	**CV, %**	**ICC**
SDNN (ms)	34.1 ± 9.6 (30.7–37.6)	33.1 ± 12.0 (28.8–37.4)	33.1 ± 10.6 (29.2–36.9)	37.5 ± 14.1 (32.4–42.5)	34.4 ± 9.9 (30.9–38.0)	6.85 (5.91–8.36)	0.68 (0.51–0.82)
RMSSD (ms)	16.0 ± 4.8 (14.3–17.7)	17.3 ± 8.9 (14.0–20.4)	16.9 ± 7.3 (14.3–19.5)	19.1 ± 8.9 (15.9–22.3)	17.3 ± 6.4 (15.0–19.6)	4.47 (3.85–5.45)	0.68 (0.51–0.82)
LF (ms)^2^	598 ± 638 (306–818)	524 ± 548 (327–721)	469 ± 305 (359–578)	577 ± 694 (327–826)	540 ± 505 (358–721)	324 (280–395)	0.69 (0.53–0.83)
LF (n.u.)	82.13 ± 20.48 (74.30–89.48)	78.91 ± 20.65 (71.50–86.33)	79.26 ± 21.09 (71.69–86.83)	78.13 ± 21.94 (70.25–86.00)	79.61 ± 19.19 (72.72–86.49)	10.45 (9.02–12.75)	0.25 (0.05–0.49)
HF (ms)^2^	106 ± 60 (85–128)	129 ± 124 (84–173)	120 ± 86 (89–151)	140 ± 125 (95–184)	124 ± 71 (98–149)	83 (72–101)	0.36 (0.15–0.58)
HF (n.u)	17.87 ± 10.34 (14.16–21.58)	21.08 ± 12.37 (16.64–25.52)	20.73 ± 12.94 (16.09–25.38)	21.86 ± 14.63 (16.61–27.12)	20.39 ± 9.25 (17.06–23.71)	10.45 (9.02–12.75)	0.25 (0.05–0.49)
SD1 (ms)	12.5 ± 6.1 (10.3–14.7)	13.6 ± 7.3 (10.9–16.2)	12.5 ± 5.6 (10.5–14.5)	13.4 ± 6.7 (11.0–15.8)	13.0 ± 5.4 (11.0–14.9)	3.98 (3.44–4.86)	0.43 (0.23–0.64)
SD2 (ms)	45.7 ± 21.1 (38.1–53.2)	46.2 ± 15.6 (40.5–51.8)	51.4 ± 19.5 (44.3–58.3)	49.7 ± 21.2 (42.0–57.2)	48.2 ± 15.5 (42.6–53.8)	13.23 (11.42–16.15)	0.56 (0.36–0.74)
α1	1.40 ± 0.24 (1.31–1.49)	1.33 ± 0.19 (1.31–1.45)	1.46 ± 0.20 (1.40–1.54)	1.46 ± 0.18 (1.40–1.53)	1.42 ± 0.17 (1.39–1.47)	19.2 (16.5–23.4)	0.14 (−0.05–0.38)
SampEn	1.30 ± 0.39 (1.16–1.44)	1.19 ± 0.36 (1.06–1.32)	1.14 ± 0.28 (1.04–1.24)	1.20 ± 0.39 (1.06–1.34)	1.21 ± 0.36 (1.13–1.29)	0.95 (0.82–1.16)	0.10 (−0.08–0.34)

*Values are mean ± SD (confidence intervals, CI 90%); standard deviation of all R-R intervals (SDNN), root mean square of successive differences between normal sinus R-R intervals (RMSSD), low-frequency (LF), high-frequency (HF), Very-low frequency (VLF), Total power, short-term beat-to-beat R-R variability from the Poincaré plot (SD1), long-term beat-to-beat variability from the Poincaré plot (SD2), and detrended fluctuations of short fractal scaling (α1), sample entropy (SampEn), TEM expressed as the coefficient of variation (CV%), intra-class correlation coefficient (ICC)*.

**Table 3:** **Heart rate variability ambulatory measures (4 h) and their corresponding reliability measures**.

	**Tuesday**	**Wednesday**	**Thursday**	**Friday**	**Average**	**CV, %**	**ICC**
SDNN (ms)	95.0 ± 29.3 (82.2–107.7)	94.9 ± 20.0 (84.8–104.9)	102.3 ± 36.7 (87.1–117.4)	101.9 ± 29.6 (88.2–108.8)	98.5 ± 20.2 (88.2–114.8)	25.7 (22.2–31.4)	0.25 (0.05–0.49)
RMSSD (ms)	26.4 ± 10.0 (22.6–30.2)	24.3 ± 10.2 (20.3–28.1)	24.7 ± 8.9 (21.0–28.3)	26.2 ± 9.8 (22.2–28.3)	25.3 ± 8.6 (21.8–28.8)	5.0 (4.3–6.1)	0.75 (0.60–0.86)
LF (ms)^2^	905 ± 413 (740–1067)	887 ± 468 (708–1071)	876 ± 412 (709–1037)	954 ± 529 (753–1160)	905 ± 428 (736–1075)	191 (165–234)	0.84 (0.73–0.91)
LF (n.u.)	80.16 ± 19.11 (73.30–87.02)	80.24 ± 19.37 (73.29–87.19)	80.62 ± 19.57 (73.60–87.65)	79.94 ± 19.33 (73.00–86.88)	80.24 ± 19.00 (73.42–87.06)	4.42 (3.81–5.39)	0.76 (0.62–0.87)
HF (ms)^2^	239 ± 172 (189–286)	234 ± 186 (168–295)	232 ± 174 (166–288)	256 ± 199 (191–338)	240 ± 168 (183–298)	81 (70–99)	0.82 (0.70–0.90)
HF (n.u.)	19.84 ± 8.30 (16.86–22.82)	19.75 ± 8.48 (16.58–22.93)	19.37 ± 9.22 (16.06–22.68)	20.05 ± 9.06 (16.80–23.30)	19.75 ± 8.09 (16.85–22.66)	4.42 (3.81–5.39)	0.76 (0.62–0.87)
SD1 (ms)	18.2 ± 7.3 (15.4–20.9)	17.5 ± 6.9 (14.7–20.1)	17.3 ± 6.2 (14.8–19.9)	18.7 ± 6.9 (15.8–21.5)	17.9 ± 6.1 (15.4–20.4)	3.5 (3.1–4.3)	0.75 (0.60–0.86)
SD2 (ms)	141.4 ± 59.5 (117.4–165.3)	131.9 ± 28.2 (117.8–146.0)	142.4 ± 51.7 (121.1–163.7)	142.9 ± 41.5 (124.7–161.0)	139.6 ± 31.5 (124.2–155.0)	39.4 (34.0–48.1)	0.30 (0.10–0.53)
α1	1.48 ± 0.12 (1.33–1.57)	1.45 ± 0.14 (1.33–1.57)	1.46 ± 0.12 (1.34–1.58)	1.44 ± 0.12 (1.32–1.56)	1.45 ± 0.11 (1.33–1.57)	0.07 (0.06–0.09)	0.69 (0.53–0.83)
SampEn	0.92 ± 0.23 (0.82–1.03)	1.01 ± 0.67 (0.76–1.27)	0.84 ± 0.19 (0.74–0.93)	0.90 ± 0.19 (0.81–1.00)	0.92 ± 0.38 (0.80–1.04)	0.95 (0.82–1.16)	0.11 (-0.07–0.35)

*Values are mean ± SD (confidence intervals, CI 90%); standard deviation of all R-R intervals (SDNN), root mean square of successive differences between normal sinus R-R intervals (RMSSD), low-frequency (LF), high-frequency (HF), Very-low frequency (VLF), Total power, short-term beat-to-beat R-R variability from the Poincaré plot (SD1), long-term beat-to-beat variability from the Poincaré plot (SD2), and detrended fluctuations of short fractal scaling (α1), sample entropy (SampEn), TEM expressed as the coefficient of variation (CV%), intra-class correlation coefficient (ICC)*.

**Table 4:** **Heart rate recovery over 5 min during 4 days and their corresponding reliability measures**.

	**Tuesday**	**Wednesday**	**Thursday**	**Friday**	**Average**	**CV, %**	**ICC**
HRend	155 ± 6 (152–157)	153 ± 4 (152–155)	155 ± 4 (153–157)	155 ± 3 (153–156)	154 ± 5 (153–156)	2.67 (2.31–3.26)	0.74 (0.59–0.85)
**HR RECOVERY**							
Δ 1′ (bpm)	36 ± 7 (33–39)	34 ± 7 (31–37)	33 ± 8 (29–35)	34 ± 11 (30–38)	34 ± 7 (32–37)	6.33 (5.46–7.72)	0.51 (0.31–0.70)
Δ 2′ (bpm)	49 ± 6 (46–51)	44 ± 9 (40–47)	46 ± 8 (43–48)	45 ± 8 (42–48)	46 ± 5 (44–48)	6.50 (5.61–7.93)	0.39 (0.18–0.61)
Δ 3′ (bpm)	51 ± 8 (48–54)	50 ± 13 (45–55)	50 ± 7 (47–52)	51 ± 9 (47–54)	50 ± 7 (48–53)	8.32 (7.18–10.15)	0.50 (0.29–0.69)
Δ 5′ (bpm)	58 ± 10 (54–61)	53 ± 8 (50–56)	56 ± 6 (54–58)	55 ± 8 (52–58)	55 ± 5 (53–57)	6.90 (5.95–8.42)	0.37 (0.16–0.59)
T30	253 ± 100 (217–288)	266 ± 100 (230–301)	226 ± 69 (201–251)	238 ± 57 (217–258)	246 ± 84 (216–275)	75.29 (64.98–91.86)	0.20 (0.01–0.44)
HRR τ (s)	67 ± 27 (54–80)	65 ± 14 (57–72)	74 ± 31 (58–89)	60 ± 18 (51–69)	67 ± 23 (56–78)	31.3 (26.3–39.7)	0.36 (0.14–0.59)

*Values are mean ± SD (confidence intervals, CI 90%); end heart rate in the test (HRend), negative reciprocal of the slope of the regression line of heart beats during the initial rapid HR decrease (from the 10th to the 40th s) (T30); heart rate change in first minute (Δ 1′), heart rate change in two first minute (Δ 2′), heart rate change in three first minute (Δ 3′), heart rate change in five first minute (Δ 5′), heart rate recovery in time constant (HRR τ). TEM expressed as the coefficient of variation (CV%), intra-class correlation coefficient (ICC)*.

### Relationships between autonomic indices and PA

**Figure 2:**
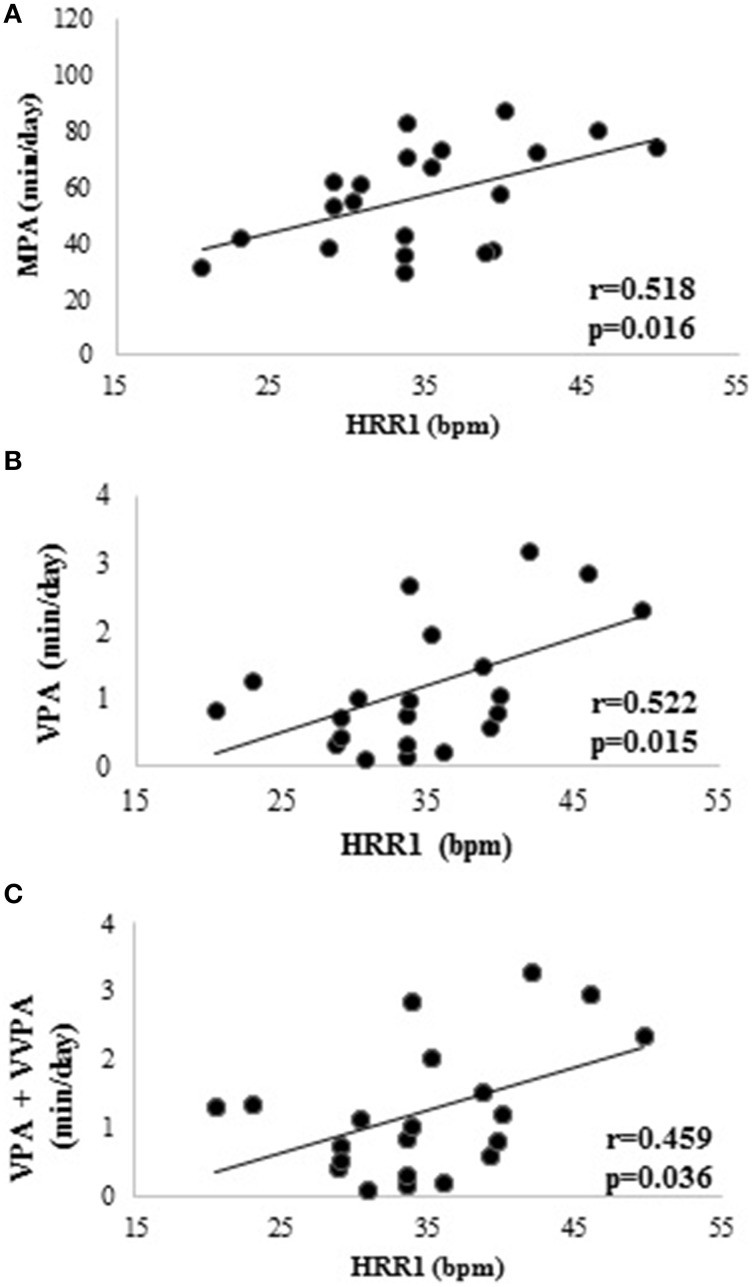
**Relationships between heart rate recovery in the first min and physical activity measures**. **(A)** Relationship between heart rate recovery in the first min and mean daily moderate physical activity. **(B)** Relationship between heart rate recovery in the first min and mean daily vigorous physical activity. **(C)** Relationship between heart rate recovery in the first min and sum of vigorous physical activity and very vigorous physical activity. HRR1, 4-day average heart rate recovery within the first minute; VPA, mean daily vigorous physical activity; VPA+VVPA, sum of vigorous physical activity and very vigorous physical activity.

When considering the 4-day average ambulatory HRV measures, correlations were observed between VPA and: RMSSD (*r* = −0.449, *p* = 0.041), HF (*r* = −0.520, *p* = 0.016), and SD1 (*r* = −0.463, *p* = 0.035); and between VPA+VVPA and: RMSSD (*r* = −0.453, *p* = 0.039), HF (*r* = −0.526, *p* = 0.014), and SD1 (*r* = −0.473, *p* = 0.030). Additionally, 4-day average HRR1 was correlated to MPA, VPA, and VPA+VVPA (see Figure 2) but not with Step·day^1^ (*r* = 0.376, *p* = 0.093). No correlations were found between HRV measures in the orthostatic test and measures of PA.

### Relationships between autonomic indices and CRF

Significant relationships were identified between CRF and 4-day average HRV measures with VO_2_max correlated to standing HF (*r* = 0.523, *p* = 0.015), standing LF (*r* = 0.550, *p* = 0.01), standing RMSSD (*r* = 0.641, *p* = 0.002), standing SDNN (*r* = 0.445, *p* = 0.043), seated SDNN (*r* = 0.475, *p* = 0.030), seated SD2 (*r* = 0.491, *p* = 0.024), and ambulatory LF (*r* = 0.554, *p* = 0.009). Average 4-day HRR measures were not correlated to CRF.

### Relationships among autonomic indices

Significant relationships were determined between the 4-day average values for HRV measures during the orthostatic test and the 4-h ambulatory period (see Table 5). Most of these correlations were moderate. No correlations were found between HRR and HRV measures.

**Table 5:** **Matrix of correlations between 4-day average values for HRV measures during the orthostatic test and 4-h ambulatory recordings**.

**Ambulatory HRV**									
		**RMSSD**	**SDNN**	**LF**	**HF**	**SD1**	**SD2**	**SampEn**	**α 1**
		***r (p)***	***r (p)***	***r (p)***	***r (p)***	***r (p)***	***r (p)***	***r (p)***	***r (p)***
RMSSD	Standing	0.602 (0.00)	0.316 (0.16)	0.651 (0.00)	0.493 (0.02)	0.602 (0.00)	0.311 (0.17)	0.522 (0.01)	−0.208 (0.20)
	Seated	0.640 (0.00)	0.203 (0.37)	0.586 (0.00)	0.524 (0.01)	0.672 (0.00)	0.199 (38)	0.297 (0.19)	−0.234 (0.07)
SDNN	Standing	0.407 (0.06)	0.262 (0.25)	0.517 (0.01)	0.701 (0.00)	0.406 (0.06)	0.257 (0.26)	0.442 (0.04)	−0.246 (0.28)
	Seated	0.598 (0.00)	0.718 (0.00)	0.733 (0.00)	0.924 (0.00)	0.596 (0.00)	0.710 (0.00)	0.385 (0.08)	−0.654 (0.00)
LF	Standing	0.651 (0.00)	0.456 (0.03)	0.570 (0.00)	0.634 (0.00)	0.719 (0.00)	0.455 (0.03)	0.372 (0.09)	−0.294 (0.10)
	Seated	0.594 (0.00)	0.386 (0.08)	0.618 (0.00)	0.338 (0.15)	0.406 (0.06)	0.383 (0.08)	0.568 (0.00)	−0.007 (0.92)
HF	Standing	0.535 (0.01)	0.308 (0.18)	0.548 (0.01)	0.547 (0.01)	0.510 (0.01)	0.299 (0.18)	0.455 (0.04)	−0.508 (0.01)
	Seated	0.541 (0.00)	0.231 (0.31)	0.388 (0.07)	0.632 (0.00)	0.577 (0.00)	0.226 (0.32)	0.348 (0.12)	−0.422 (0.05)
SD1	Standing	0.602 (0.00)	0.386 (0.08)	0.751 (0.00)	0.480 (0.02)	0.596 (0.00)	0.383 (0.08)	0.471 (0.03)	−0.222 (0.33)
	Seated	0.641 (0.00)	0.634 (0.00)	0.615 (0.00)	0.597 (0.00)	0.926 (0.00)	0.626 (0.00)	0.481 (0.02)	−0.753 (0.00)
SD2	Standing	0.733 (0.00)	0.570 (0.00)	0.569 (0.00)	0.615 (0.00)	0.773 (0.00)	0.564 (0.00)	0.276 (0.22)	−0.081 (0.72)
	Seated	0.460 (0.03)	0.456 (0.04)	0.680 (0.00)	0.405 (0.07)	0.461 (0.04)	0.455 (0.04)	0.389 (0.08)	−0.055 (0.81)
SampEn	Standing	0.357 (0.11)	−0.047 (0.84)	0.409 (0.06)	0.309 (0.17)	0.361 (0.10)	−0.060 (0.79)	0.314 (0.16)	−0.175 (0.44)
	Seated	0.585 (0.00)	0.313 (0.16)	0.404 (0.07)	0.511 (0.01)	0.557 (0.00)	0.327 (0.14)	0.527 (0.01)	−0.583 (0.00)
α 1	Standing	0.562 (0.00)	0.303 (0.18)	0.643 (0.00)	0.480 (0.03)	0.562 (0.00)	0.299 (0.19)	−0.211 (0.36)	−0.236 (0.30)
	Seated	0.672 (0.00)	0.250 (0.27)	0.570 (0.00)	0.597 (0.00)	0.673 (0.00)	0.245 (0.28)	−0.079 (0.73)	−0.466 (0.03)

*Root mean square of successive differences between normal sinus R-R intervals (RMSSD), Standard deviation of all R-R intervals (SDNN), absolute low-frequency (LF), absolute high-frequency (HF), short-term beat-to-beat R-R variability from the Poincaré plot (SD1), long-term beat-to-beat variability from the Poincaré plot (SD2), sample entropy (SampEn), and detrended fluctuations of short fractal scaling (α1)*.

No correlations were identified between CRF and levels of PA.

## Discussion

The current study has confirmed that cardiac autonomic indices were associated with PA and CRF in a group of healthy, overweight women who did not exercise with different relationships identified based upon the HRV condition (i.e., seated, standing, or ambulatory). Specifically, greater measures of HRV were associated with greater CRF. Similarly greater HRR1 was associated with greater PA including MPA, VPA, and VVPA. Therefore, CRF may be a more important influence than PA in enhancing HRV while PA may be integral for enhancing parasympathetic reactivation. Given the low-to-moderate levels of reliability exhibited by these HR measures, the use of average weekly recordings in further studies is recommended for a more precise evaluation of autonomic control of HR.

The most novel finding of the current study was the moderate relationships between PA measures and the 4-day average of HRR1. To our knowledge, this is the first time a relationship between objectively measured PA levels and parasympathetic reactivation has been reported and reinforces the important role that incidental PA has on the autonomic control of HR and potential cardiovascular health (Cole et al., 1999). This relationship was identified in a unique sample of overweight women who did not exercise and clearly highlights the impact of incidental PA on HR control unlike previous studies evaluating the relationship between PA levels and autonomic indices (Buchheit et al., 2005, 2006; Buchheit and Gindre, 2006). Previous studies failed to differentiate between incidental and exercise based PA (Buchheit et al., 2005, 2006) and employed questionnaires for the identification of training load (Buchheit and Gindre, 2006). These factors may have masked potential associations between PA and HR control with further studies warranted to verify this relationship in other populations of different gender and age.

In line with a previous report (Hautala et al., 2010), we observed negative relationships between short-term, vagally related HRV measures (e.g., RMSSD, HF, and SD1) during ambulatory conditions and objectively measured PA levels. This was an expected finding that reinforces the robustness of long-term HRV measures (i.e., SDNN and SD2) for autonomic evaluations in ambulatory conditions as these measures were unaffected by PA levels. Further, these results may indirectly reflect the relationship between CRF, PA, and HRV with those adults undertaking more PA likely to exhibit greater CRF and subsequently higher HRV. While we did not find any relationship between PA and CRF, maybe because of the low levels of VPA and VVPA recorded, further longitudinal interventions may elucidate the possible influence of greater levels of incidental PA on CRF in different populations.

In our study, a greater CRF (i.e., VO_2_max) was positively related to greater weekly average HRV levels during a range of conditions (e.g., postures, ambulation). These moderate relationships were in line with previous studies that reported a greater autonomic control of HR in those individuals with greater CRF or running capacity (Buchheit and Gindre, 2006; Kiviniemi et al., 2007; Hautala et al., 2009; Boullosa et al., 2013). However, and in contrast to previous studies (Boullosa et al., 2009; Daanen et al., 2012), no relationship was observed between CRF and HRR measures. Although speculative, this lack of significant correlation could reflect the homogeneous sample of women that were not engaged in regular exercise (i.e., low VO_2_max). In addition, the absence of correlations between CRF and HRR measures could reflect a differential regulation of HRR in females with gender differences in post-exercise, autonomic control previously reported (Mendonca et al., 2010; Barak et al., 2014). Further, the females undertook incidental PA of varying intensities that may have impacted HRR in a similar way that training load impacted on HRR in previous studies with regular exercisers (Buchheit and Gindre, 2006; Guerra et al., 2014). This consideration is important as previous studies were conducted within sport settings and participants with high CRF. In contrast, the current study has been the first to our knowledge, to report these relationships within a work environment (i.e., non-athletes). Overall, these findings highlight the various contributors (i.e., exercise and incidental PA) to different cardiac autonomic indices and reinforce the value of considering both HRV and HRR when evaluating the autonomic control of HR and its relationship with CRF in different populations. The exact contributions of incidental PA and exercise-based PA to cardiac autonomic control in physically active individuals remains to be differentiated in future studies.

The relationships between average HRV measures during the orthostatic test and the averaged ambulatory HRV measures (Table 5) were primarily moderate with long-term HRV measures unrelated to ambulatory measures. Previously, long-term HRV indices were reported to be more robust during ambulatory conditions (Hautala et al., 2010). Within the current study, short-term (i.e., RMSSD, SD1, and α1) and frequency domain (i.e., HF and LF) HRV measures exhibited better absolute and relative reliability during ambulatory conditions (i.e., 4 h) when compared to shorter recordings (i.e., 2 min) in seated and standing postures. The moderate reliability for a range of HRV measures reinforces the variable nature of HRV over a normal week with special attention to the reliability of HRV parameters in different conditions encouraged for better assessments of cardiac autonomic adaptations. Finally, the absence of a relationship between HRR and HRV was in agreement with a previous study of healthy individuals (Esco et al., 2010), and reinforces the specific and unique contributors to these different autonomic measures.

The cross-sectional design of the current study suggests some caution and the necessity of further longitudinal studies for verifying the direction and strength of the relationships between these health related parameters. Additionally, our findings were limited to adult female workers with further studies of other populations needed for elucidating the possible role of age on these relationships. Of note, recent evidence (Triggiani et al., 2015) suggests that HRV seems to be reduced in overweight healthy adult women therefore further studies should verify if the current results would be different in males and females with different percentages of body fatness. Finally, different protocols for HRV and HRR evaluations were conducted with further studies encouraged to employ other recent protocols such as recording HR measures during walking at a fixed speed (Boullosa et al., 2014) and ultra-short term HR measures (Ostojic et al., 2010; Nakamura et al., 2015).

In conclusion, the current study has defined the relationships between cardiac autonomic indices and PA and CRF in a group of healthy, non-exercising overweight women. Greater HRV was associated with greater CRF that highlighted the merit of improving CRF as a means to enhance cardiac autonomic control. In contrast, greater post-exercise HRR was associated with greater PA that reflects the unique pertinence of PA for enhancing parasympathetic reactivation. Finally, relationships between cardiac autonomic indices and other health indicators (PA and CRF) were influenced by the type and frequency of measure utilized with further studies recommended for an enhanced understanding of the contributors to autonomic control of HR for health.

## Author contributions

LT, study design, data collection, data analysis and interpretation of the results, writing of the manuscript. FR, study design, data analysis and interpretation of the results, writing of the manuscript. IO, data analysis and interpretation of the results, writing of the manuscript. SD, data analysis and interpretation of the results, writing of the manuscript. AL, data analysis and interpretation of the results, writing of the manuscript. DB, study design, data analysis and interpretation of the results, writing of the manuscript.

### Conflict of interest statement

The authors declare that the research was conducted in the absence of any commercial or financial relationships that could be construed as a potential conflict of interest.
